# Survival prediction of glioblastoma patients using modern deep learning and machine learning techniques

**DOI:** 10.1038/s41598-024-53006-2

**Published:** 2024-01-29

**Authors:** Samin Babaei Rikan, Amir Sorayaie Azar, Amin Naemi, Jamshid Bagherzadeh Mohasefi, Habibollah Pirnejad, Uffe Kock Wiil

**Affiliations:** 1 Department of Computer Engineering, Urmia University, Urmia, Iran; 2 SDU Health Informatics and Technology, The Maersk Mc-Kinney Moller Institute, University of Southern Denmark, Odense, Denmark; 3 Erasmus School of Health Policy and Management (ESHPM), Erasmus University Rotterdam, Rotterdam, The Netherlands; 4 Patient Safety Research Center, Clinical Research Institute, Urmia University of Medical Sciences, Urmia, Iran

**Keywords:** Disease-free survival, Data mining, Data processing, Machine learning, Predictive medicine, Cancer models, Head and neck cancer

## Abstract

In this study, we utilized data from the Surveillance, Epidemiology, and End Results (SEER) database to predict the glioblastoma patients’ survival outcomes. To assess dataset skewness and detect feature importance, we applied Pearson's second coefficient test of skewness and the Ordinary Least Squares method, respectively. Using two sampling strategies, holdout and five-fold cross-validation, we developed five machine learning (ML) models alongside a feed-forward deep neural network (DNN) for the multiclass classification and regression prediction of glioblastoma patient survival. After balancing the classification and regression datasets, we obtained 46,340 and 28,573 samples, respectively. Shapley additive explanations (SHAP) were then used to explain the decision-making process of the best model. In both classification and regression tasks, as well as across holdout and cross-validation sampling strategies, the DNN consistently outperformed the ML models. Notably, the accuracy were 90.25% and 90.22% for holdout and five-fold cross-validation, respectively, while the corresponding R^2^ values were 0.6565 and 0.6622. SHAP analysis revealed the importance of age at diagnosis as the most influential feature in the DNN's survival predictions. These findings suggest that the DNN holds promise as a practical auxiliary tool for clinicians, aiding them in optimal decision-making concerning the treatment and care trajectories for glioblastoma patients.

## Introduction

Glioblastomas are the most aggressive brain tumors that account for 12–15% of all brain tumors. They are the most common malignant brain tumors in adults in the United States (US) that have an incidence rate of 3.21 per 100,000^1^. Early detection and traditional treatments of glioblastomas are infrequently effective^2^ because they are invasive, and the blood–brain barrier precludes medicines from eradicating tumor cells^3^. Although the implementation of temozolomide and radiotherapy has improved glioblastoma patients' survival^4,5^, their median survival ranges from 9 to 16 months, depending on the medical care they receive^6^.

Prognostic predictions of cancer, especially glioblastoma, due to low median survival, are vital in planning patients’ treatment. Survival prediction of patients helps clinicians make informed decisions about the treatment methods and surgeries and select the most effective ones. In addition, it enables patients and their families to better comprehend the patients' condition, make appropriate decisions, and reduce their anxiety. These factors have led to the prediction of survival becoming a significant problem that requires more attention as well as accurate solutions.

The use of machine learning (ML) and deep learning (DL) methods in bioinformatics and medicine has dramatically increased^7^. They have been widely used in oncology as well and have shown promising results^8,9^. In recent years, various studies have used different ML and statistical models to predict glioblastoma patients' survival^3,10–13^. For instance, Li et al.^3^, developed nomograms to predict the survival of glioblastoma patients using SEER database. They used Cox proportional risk regression model to analyze the prognostic factors of patients and construct the nomogram. They used the effective factors for the prognosis of glioblastoma patients that Cox proportional risk regression model obtained in constructing the nomogram. The results of the nomogram revealed that the C-index of the training and verification group was 0.729 and 0.734, respectively. Al-Husseini et al.^10^ aimed to assess how a prior malignancy affects glioblastoma patients’ survival. They used the multivariable covariate-adjusted Cox models and the unadjusted Kaplan–Meier test to calculate the glioblastoma-specific and overall survival of these patients. The unadjusted Kaplan–Meier test revealed that a prior history of cancer had an adverse effect on glioblastoma-specific and overall survival for patients, and the multivariable covariate-adjusted Cox models did not show considerable differences in glioblastoma-specific or overall survival. Senders et al.^11^ proposed both statistical and ML algorithms and developed online software to predict the number of survival months (regression) and 1-year survival status (binary classification) of glioblastoma patients based on SEER. Three statistical models were utilized in their study. The C-index values of their accelerated failure time model in predicting the overall survival and 1-year survival status were 0.70 and 0.70. Samara et al.^12^ introduced an ensemble learning model to predict glioblastoma patients' survival based on SEER database. They used four ML algorithms as base classifiers and four ensemble techniques. RF achieved the best results. They reported area under the curve (AUC) values of 0.937, 0.780, and 0.893 for short-, intermediate- and long-term survival, respectively. Bakirarar et al.^13^ used five ML and two hybrid models consisting of ML models that they created to predict the 2-year and 1-year survival of glioblastoma patients based on SEER database. The hybrid models achieved the best results with for AUC metric as 0.856 and 0.764 for 1-year and 2-year survival, respectively.

Nonetheless, notable research gaps persist in existing studies. While DL has demonstrated promising outcomes in predicting survival for cancer patients^14–17^, none of the preceding investigations, to the best of our knowledge, have harnessed DL algorithms to predict the survival of glioblastoma patients using the Surveillance Epidemiology and End Results (SEER) database—an invaluable resource in the realm of cancer research^11–13,18–21^. Additionally, the majority of SEER-based studies have predominantly approached the glioblastoma survival prediction problem through a binary lens, focusing on whether a patient will survive for a specific period^11–13^. This limited perspective overlooks the potential insights offered by multiclass classification and regression approaches. Furthermore, these studies have largely neglected the critical aspect of model explainability and interpretability. The absence of attention to these factors diminishes the trust clinicians may place in ML and DL) models in real-world practice.

In this study, we address these gaps by developing five ML models—extreme gradient boosting (XGBoost), adaptive boosting (AdaBoost), decision tree (DT), K-nearest neighbors (KNN), and random forest (RF)—as well as a deep neural network (DNN) model. Our aim is to predict glioblastoma patients' survival using both classification and regression approaches. This study marks three significant contributions:

### Pioneering use of DL in glioblastoma survival prediction

This study stands as the first to use DL in both classification and regression approaches to predict glioblastoma patients' survival based on the SEER database. By evaluating the performance of both ML and DL models, it provides a more comprehensive understanding of their capabilities.

### Clinically meaningful survival classes

Departing from binary classification, this study introduces and utilizes five clinically meaningful classes for survival based on established clinical guidelines. This innovative approach aims for more accurate predictions, facilitating effective and precise treatment planning for glioblastoma patients by predicting the expected duration of survival.

### Enhanced model interpretability with SHAP

A pioneering effort, this study utilizes Shapley Additive Explanations (SHAP) to interpret SEER-based survival predictions for glioblastoma patients. This not only contributes to the transparency of our study but also enhances its reliability and robustness, making strides toward building trust in the utilization of ML and DL models in clinical decision-making.

## Results

According to the ordinary least squares (OLS) method^22,23^, as can be seen from Table S1 in the Supplementary, all features except two features (sex and marital status at diagnosis) had a significant relation with the predicted survival at the level of 0.05. However, these two features were also used in this study, because they have been used in all similar studies, and they are clinically meaningful^3,10–12,19,20^.

The results of Pearson's coefficient for skewness^24^ showed that initially, the classification dataset was moderately skewed, and the regression dataset was highly skewed, with skewness values of + 0.65 and + 2.42, respectively. After applying Synthetic Minority Oversampling Technique (SMOTE)^25,26^, the skewness value of the classification dataset reached + 0.08, which indicates it has become fairly symmetrical. Also, after applying Synthetic Minority Over-Sampling with Gaussian Noise (SMOGN)^27^, the skewness value of the regression dataset reached + 1.00, which became moderately skewed since the value is between ± 0.5 and ± 1.

### Proposed classifiers results

**Figure 1:**
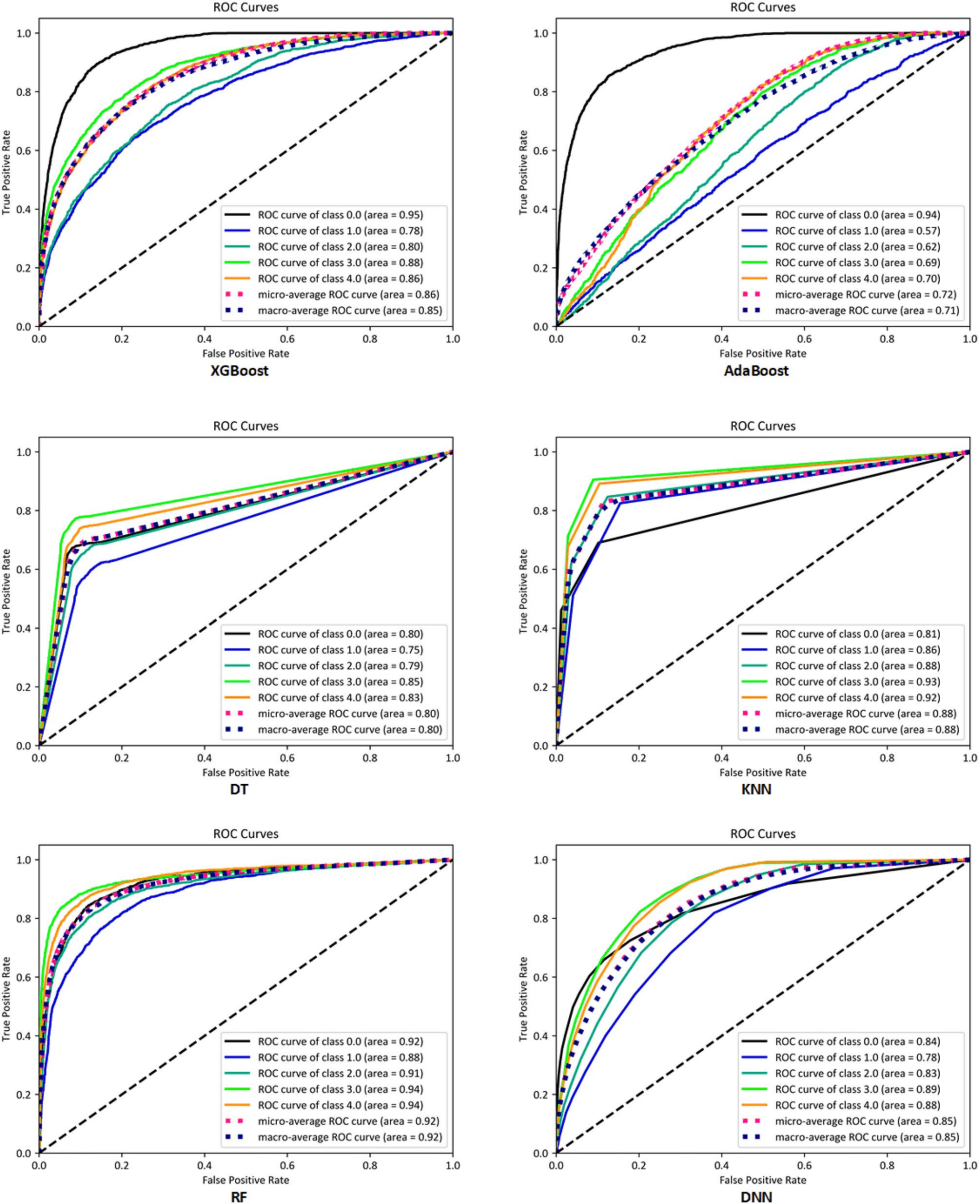
AUC diagrams of the proposed models in holdout strategy. In each class, different models have different AUC values. RF has the highest average AUC.

**Figure 2:**
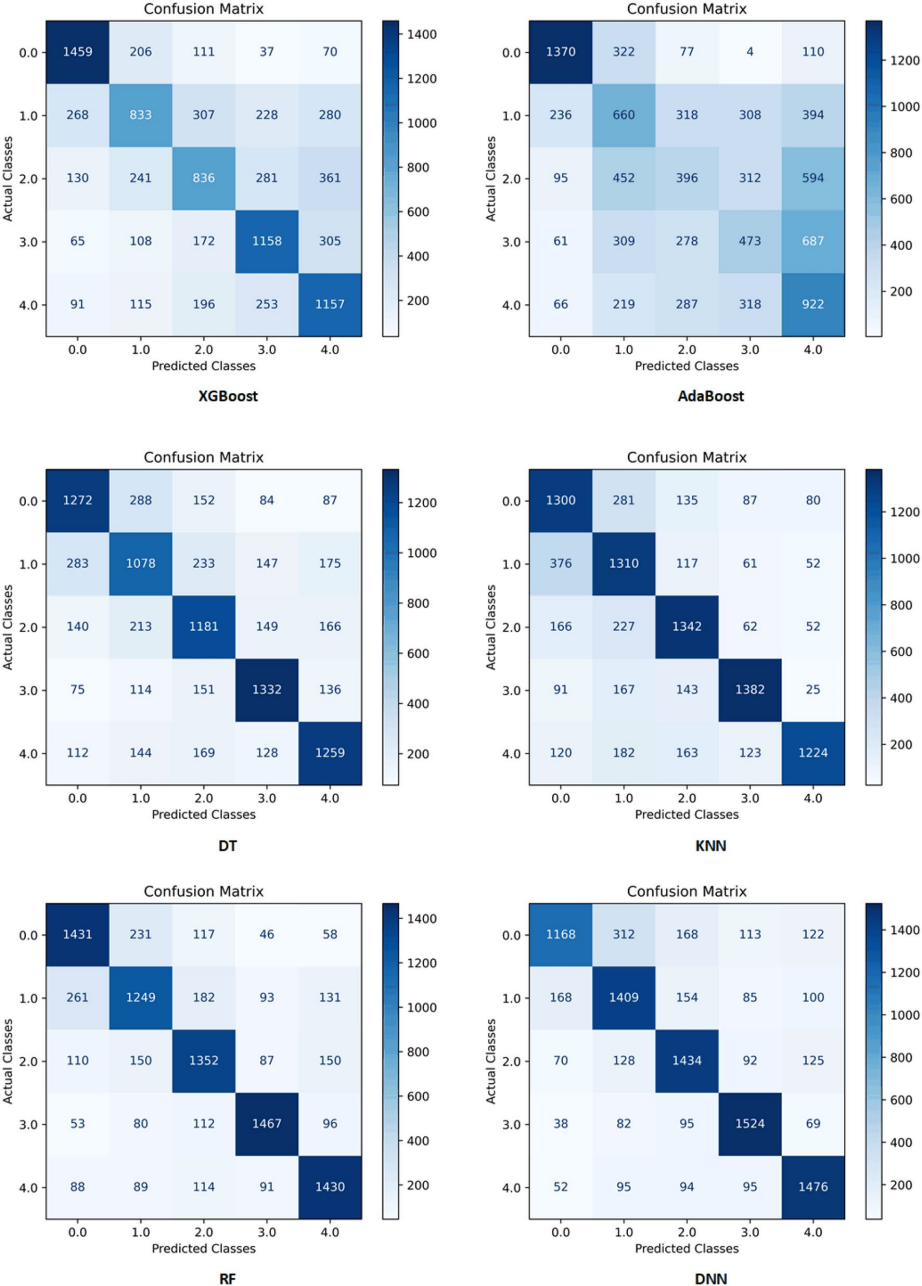
Confusion matrices of the proposed models in holdout strategy shows that DNN is the best model and has the lowest error rate.

The classification models’ results for each class in holdout and five-fold cross-validation sampling strategies are presented in Tables 1 and S2 in Supplementary, respectively. The AUC diagrams^28,29^ and confusion matrices^30,31^ of all the models in holdout sampling strategy are presented in Figs. 1 and 2, respectively. Also, in five-fold cross-validation sampling strategy, the AUC diagrams and confusion matrices of all folds of DNN are presented in Figs. S1 and S2 in the Supplementary, respectively.

**Table 1:** The proposed models’ performance for the classification approach in holdout strategy.

Model	Class	Accuracy (%)	F1-score (%)	Specificity (%)	Sensitivity (%)	AUC
XGBoost	Class 0	89.44	**74.89**	92.49	**77.48**	**0.95**
AdaBoost	Class 0	89.52	73.83	93.79	72.75	0.94
DT	Class 0	86.82	67.57	91.74	67.55	0.80
KNN	Class 0	85.58	66.05	89.80	69.03	0.81
RF	Class 0	**89.59**	74.80	93.06	75.99	0.92
DNN	Class 0	88.74	69.13	**95.55**	62.02	0.84
XGBoost	Class 1	81.08	48.72	90.88	43.47	0.78
AdaBoost	Class 1	72.40	34.03	82.29	34.44	0.57
DT	Class 1	82.76	57.44	89.67	56.26	0.75
KNN	Class 1	84.21	64.16	88.34	68.37	0.86
RF	Class 1	86.86	67.24	**92.51**	65.18	**0.89**
DNN	Class 1	**87.87**	**71.48**	91.60	**73.53**	0.78
XGBoost	Class 2	80.58	48.17	89.40	45.21	0.80
AdaBoost	Class 2	73.96	24.71	87.06	21.41	0.62
DT	Class 2	85.18	63.24	90.49	63.87	0.79
KNN	Class 2	88.50	71.59	92.47	72.58	0.88
RF	Class 2	88.97	72.57	92.92	73.12	**0.91**
DNN	Class 2	**90.00**	**75.59**	**93.11**	**77.55**	0.83
XGBoost	Class 3	84.36	61.51	89.29	64.04	0.88
AdaBoost	Class 3	75.43	29.35	87.37	26.16	0.69
DT	Class 3	89.38	73.02	93.19	73.67	0.85
KNN	Class 3	91.81	78.45	95.53	76.43	0.93
RF	Class 3	**92.90**	81.68	**95.75**	81.13	**0.94**
DNN	Class 3	92.78	**82.00**	94.83	**84.29**	0.89
XGBoost	Class 4	81.97	58.06	86.37	63.85	0.86
AdaBoost	Class 4	71.13	40.80	76.06	50.88	0.70
DT	Class 4	87.94	69.27	92.43	69.48	0.83
KNN	Class 4	91.40	75.43	**97.19**	67.55	0.92
RF	Class 4	91.18	77.78	94.16	78.91	**0.94**
DNN	Class 4	**91.88**	**79.69**	94.42	**81.45**	0.88
XGBoost	Average	83.48	58.27	89.68	58.81	0.85
AdaBoost	Average	76.48	40.54	85.31	41.12	0.71
DT	Average	86.41	66.10	91.50	66.16	0.80
KNN	Average	88.30	71.13	92.66	70.79	0.88
RF	Average	89.90	74.81	93.68	74.86	**0.92**
DNN	Average	**90.25**	**75.57**	**93.90**	**75.76**	0.85

Significant values are in [bold].

As seen in Tables 1 and S2 in the Supplementary, DNN^32,33^ achieved the highest accuracy, F1-score, specificity, and sensitivity in both holdout and five-fold cross-validation strategies on average^34,35^. RF^36^ also achieved the highest AUC on average and was our second-best model that had a very close performance to DNN in both sampling strategies.

**Figure 3:**
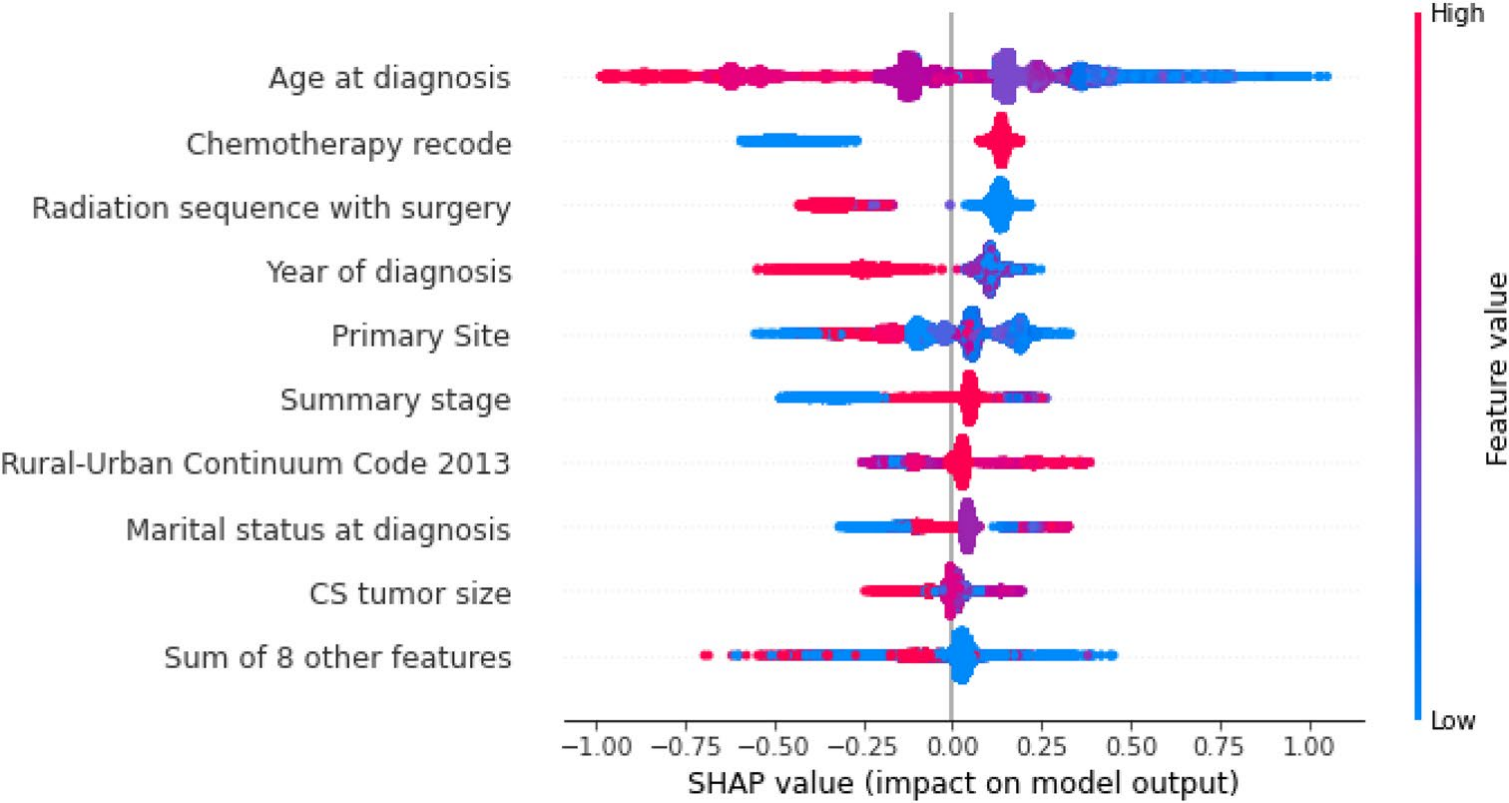
Effect of nine important features on DNN model’s output. In this plot, each point in the plot indicates a row of the dataset. The blue color indicates a lower value, and the red indicates a higher value of a feature. The distribution of the blue and red points generally indicates the directionality impact of the features. For instance, the higher value of age at diagnosis has a lower SHAP value (i.e., negative contribution), and the lower value of age has a higher SHAP value (i.e., positive contribution) on the prediction value (i.e., number of survival months).

**Figure 4:**
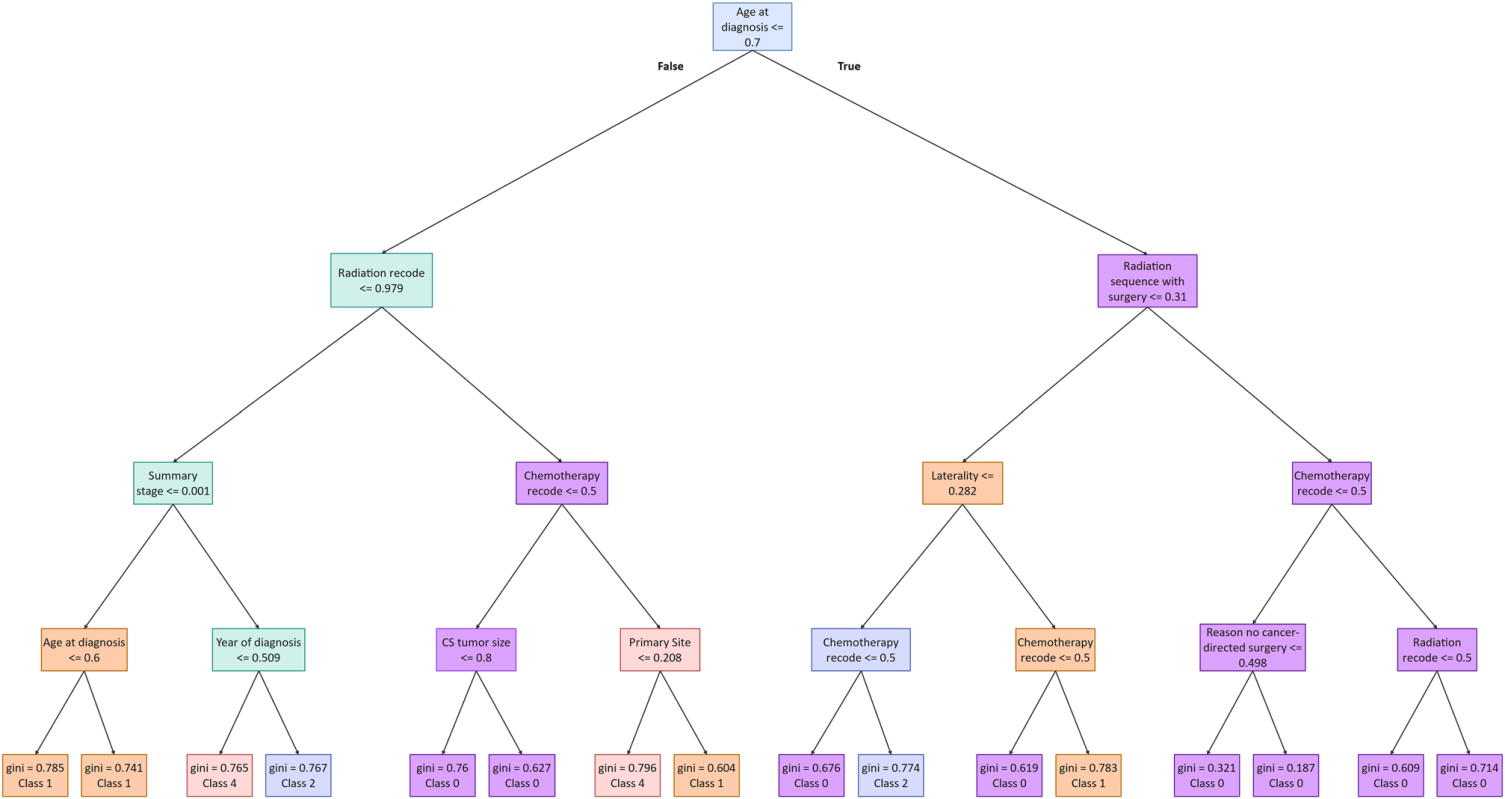
12th tree of the RF model. This tree is one of the four drawn trees whose root is age at diagnosis-the most important feature according to SHAP.

Using SHAP library^37^, the most important features in the best model (DNN) were extracted and shown in Fig. S3 in the Supplementary. The information about the weights of the nine important features on the output of the DNN model is provided in Fig. 3, which is plotted using SHAP. As shown in Fig. 3, "age at diagnosis" impacts our model's output the most. It is the most important feature with the highest SHAP value in predicting the survival of glioblastoma patients. The 12th, 17th, 63rd, and 81st trees of our RF model with depth four were randomly picked to show the predicted classes. The 12th tree is shown in Fig. 4 and the other trees were shown in Figs. S4–S6 in the Supplementary, respectively. Interestingly, in two of four trees, the root of trees is age at diagnosis, the feature with the highest SHAP value. The roots of the other two randomly selected trees of RF model are chemotherapy recode (second highest SHAP value) and year of diagnosis (fourth highest SHAP value), respectively.

### Proposed regressors results

Along with classifiers, regressors were also developed to evaluate ML and DL models more comprehensively. Mean Squared Error (MSE), Root Mean Square Error (RMSE), and Coefficient of determination (R^2^) were calculated to evaluate the performance of our regression models^38–40^. The results of holdout strategy are shown in Table 2 and the results of five-fold cross-validation strategy are represented in Table S3 in the Supplementary. As seen in both of these Tables, DNN achieved the best performance compared to the six proposed models for all the criteria. The performance of RF is also promising and very close to DNN.

**Table 2:** The proposed models’ performance for the regression approach in holdout strategy.

Model	MSE	RMSE (%)	R^2^
XGBoost	0.0168	12.96	0.6475
AdaBoost	0.0385	19.62	0.1923
DT	0.0214	14.65	0.5493
KNN	0.0317	18.03	0.3179
RF	0.0167	12.94	0.6484
DNN	**0.0163**	**12.79**	**0.6565**

Significant values are in [bold].

## Discussion

In this study, five ML models and one DL model were developed to predict the survival of glioblastoma patients using regression and classification approaches. To the best of our knowledge, this is the first SEER-based study that uses DL and multiclass ML models to predict the survival of glioblastoma patients. To make the survival intervals more meaningful for clinicians^41^, instead of binary classification, five classes of survival were selected. Moreover, SHAP was used to interpret the models’ decision-making and identify the most important features.

We used two sampling strategies of holdout and five-fold cross-validation to evaluate our models’ performance. Our results showed that in the classification approach, using both sampling strategies, when the survival is less than 6 months, XGBoost showed the best performance. However, in predicting survival when it was more than 6 months, DNN had the best performance based on confusion matrix criteria (accuracy, F1-score, sensitivity, and specificity). However, if we consider AUC criterion, RF model had the best performance among the other algorithms in predicting the survival of the patients when the survival is greater than 6 months in both holdout and five-fold cross-validation. Concordance index (C-index), a well-known measure for evaluating predictive models in healthcare, estimates the probability of concordance between observed and predicted outcomes^42,43^. Given the fact that C-index is almost identical to AUC^44^, our finding in this study is in line with the results of Mandrekar's study, and AUC of RF is considered outstanding^29^. Furthermore, it is worth mentioning that when we consider five-fold cross validation strategy, DNN showed a promising performance in predicting the classes in all five folds based on confusion matrix criteria. The insignificant difference between the results obtained by DNN in the two sampling strategies indicated that our best model perform well under different conditions.

SHAP analysis showed that age at diagnosis, chemotherapy recode, and radiation sequence with surgery are the most important features in DNN. The importance and the effects of those three features in the survival of glioblastoma patients have already been emphasized in different clinical studies^45–48^ which is also in line with our findings. For example, our SHAP diagram shows that a higher age at diagnosis has a negative contribution to survival, while a lower age has a positive contribution to the number of survival months. This is exactly in line with previous studies that stated the hazard ratio of death in patients with glioblastoma is increased with aging^49,50^. Moreover, using interpretable methods such as SHAP enables us to explain and comprehend the model's performance and decisions much better. Furthermore, they help ML models obtain more comprehensible results than statistical models^51^. In half of the drawn DTs from the RF model, the root of the tree was age at diagnosis, and this also proved the importance of this feature in predicting glioblastoma patients’ survival. Similarly, in the regression approach, DNN achieved the best results in all criteria. The second-best model by a small margin was RF. The regressors predicted the exact number of months patients would live, which can help to plan the treatment of patients precisely*.*

Table 3 compares the best classification results of two other studies with our study. As shown in the table, although the survival problem was considered a non-binary problem, the performance of the best model for classification was better compared to the binary approach taken by the presented other studies and had an excellent AUC according to Mandrekar^29^.

**Table 3:** Comparison of the performance of this study and previous studies in classification approach.

Study	Method	Class selection		Accuracy	F1-Score	AUC
Samara et al.^12^	RF	Binary (Studies that have performed binary classification predicted whether the patient would survive for specified periods or not.)	Short-term survival	0.86	–	0.937
			Intermediate-term survival	0.70	–	0.780
			Long-term survival	0.81	–	0.893
Bakirar et al.^13^	Hybrid model (J48, Multi-Layer Perceptron and Naïve Bayes for 1-year survival and J48, Multi-Layer Perceptron and Logistic Regression for 2-year survival)	Binary	1-year survival	0.849	–	0.856
			2-year survival	0.741	–	0.764
This study	DNN (holdout)	Non-binary (5 classes)		0.9025	0.77	0.85
	DNN (five-fold cross-validation)			0.9022	0.75	0.84

It's important to acknowledge the limitations of this study, as they impact the interpretation of results. In particular, the absence of comprehensive treatment details, such as the sequencing of treatment procedures, additional comorbid conditions like diabetes^52^, and even genetic profiles of the patients represents constraints. Incorporating such information, obtainable from hospital records or insurance databases, could enhance the dataset, leading to more accurate survival predictions and a more thorough evaluation of the models' predictive capabilities. Moreover, the current models lack external validation, as an independent dataset for validation purposes was unavailable. To address this limitation, future endeavors aim to collect an external dataset similar to the current one. The validation of the best models against this new dataset would support the robustness and reliability of the study, demonstrating the generalizability of the models to different data contexts.

In conclusion, despite these limitations, the DNN model exhibited exceptional performance in both classification and regression approaches in two different sampling strategies, positioning it as a valuable auxiliary tool in clinical practice. The application of this tool could empower clinicians in devising tailored treatment plans for glioblastoma patients, optimizing resource allocation, time management, and ultimately alleviating the challenges and anxieties faced by patients.

## Materials and methods

### Dataset

SEER is a source of cancer incidence and survival information that collects cancer data from 18 states in the US^53^. This database covers approximately 48.0 percent of the US population. It is one of the largest and most comprehensive databases of cancer patients in the US^54^. It provides anonymized data on patient demographics, primary tumor site, tumor morphology, the first course of treatment, stage at diagnosis, and vital status of patients. The data collected by the SEER program are accurate and complete on all cancers and in all regions. It employs a continuous quality control and improvement program that minimizes and corrects errors and ensures the data’s high quality^55^. The glioblastoma patients’ data between 2007 and 2016 was picked from SEER for this study.

### Data preprocessing

To prepare the dataset, with the help of clinical researchers, 48 features of the initial dataset were reviewed. The features that were not related to glioblastoma patient's survival were removed by clinical researchers, and those that could influence the prediction of the patients' survival were left. Afterwards, since the missing data was completely random, records with more than 30% missing value were removed from these features. It is worth mentioning that, based on^56,57^ this threshold does not introduce bias. Finally, patients who died because of other diagnoses rather than glioblastoma and those with unknown survival time were excluded. Our final dataset included 19,564 samples and 17 numerical and categorical features. The categorical features were converted to numerical with the help of clinicians. The retained features are shown in Table S4 in the Supplementary.

OLS is a common technique that is used in linear regression models. In this study, it was used to find the significant difference between features and the outcome. It defines the relationship between a dependent and one or more independent quantitative features. It shows the statistically significant differences between the features' values^22,23^. We considered the statistical significance (*p*-value) at the level of 0.05 in this test.

The target variable, the patients' survival, is defined as the number of months from diagnosis to death event. For the classification approaches, five clinically relevant classes as Class 0 (≤ 6 months), Class 1 (7–12 months), Class 2 (13–18 months), Class 3 (19–24 months), and Class 4 (≥ 25 months) were considered. For regression, the number of months that patients survived was used as the target variable.

We utilized the method of Min–Max normalization from the Sklearn library of Python to standardize the data scale and change the boundaries in the range of (0, 1) according to Eq. (1), where ${X}_{max}$ and ${X}_{min}$ denote the maximum and minimum data values, respectively.

1 $${X}_{normalize}=\frac{X-{X}_{min}}{{X}_{max}-{X}_{min}}$$

### Feature importance

The interpretability of ML models is very important to understand and trust the decision-making process. Knowing which features have more significant impacts on the model makes it easier for clinicians to interpret the performance of the model and patterns of data. As a result, medical decisions and patients' treatment processes are improved^58^. As in other medical fields, this can be useful in predicting patients' survival and help clinicians make better decisions by understanding how the model works^59,60^. Therefore, to identify each feature's impact on our models' decision-making, we used the SHAP library of Python 3.7^37^.

### Data imbalance

One of the inescapable challenges of medical datasets is data skewness because it may make sampling of the target feature non-uniform and reduce the generalizability of the model. We used Pearson's coefficient of skewness (second method) to detect the datasets’ skewness. To handle the problem of data skewness, we used SMOTE^25,26^ and SMOGN^27^ to balance the dataset in classification and regression approaches, respectively. Eventually, the final dataset for the classification had 46,340 cases, and the final dataset for regression had 28,573 cases. The distribution of the classification and regression datasets before and after balancing is shown in Fig. S7 in the Supplementary. We also used Pearson's second skewness coefficient test to show the symmetry of possible distribution in the datasets according to Eq. (2), where x ®, m, and s denote the mean, the median, and the standard deviation of our dataset, respectively^24^.

2 $$Skewness=\frac{3\times \left(\overline{x}-m\right)}{s}$$

**Figure 5:**
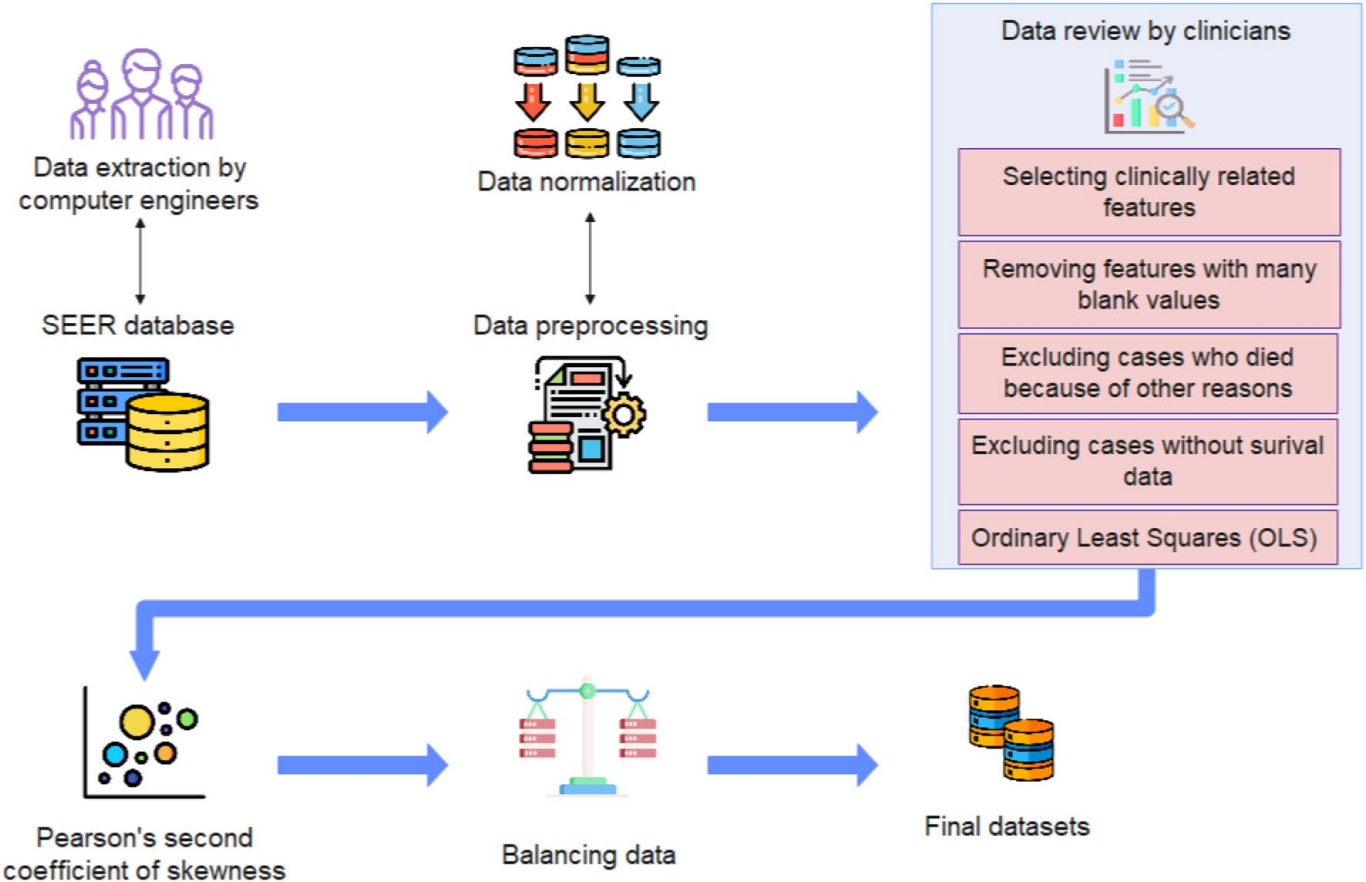
Diagram of the steps of data sets’ preparation.

The steps of the preparation of the datasets used in this study are illustrated in Fig. 5.

### Predictive models development

We developed five ML models including XGBoost, AdaBoost, DT, KNN, and RF^36^ because of their different methodologies, and a DNN model for regression and classification to predict the survival of glioblastoma patients^32,33^. The structure of our DNN model for classification is represented in Fig. S8 in the Supplementary, and for the regression model is represented in Fig. S9 in the Supplementary. In order to consider this problem as a multiclass (not binary) prediction problem, we considered five classes of survival months and predicted them using classification models. Moreover, we predicted the survival of glioblastoma patients more accurately using regression and predicted the number of months the patients would survive.

In this study, we used 32 GB RAM, Intel Xeon E5-2650 CPU, and 4 GB GPU Nvidia GTX1650, to implement our proposed models. We represented the ranges of the hyper-parameters for classification and regression models in Table S5 in the Supplementary. We obtained the optimal hyper-parameters using the GridSearchCV method and provided the hyper-parameters of both approaches in Table S6 in the Supplementary. As a result of using this method, we achieved optimal performance for both approaches.

### Data sampling strategy

In this study, the hold-out split dataset strategy is implemented, dedicating 80% of the data for training and reserving 20% for testing for both classification and regression models. Moreover, five-fold cross-validation was employed to evaluate the performance of all the developed models both in classification and regression. 80% and 20% of the dataset were apportioned for training and testing in each iteration, respectively.

### Predictive models evaluation

One common evaluation criterion in ML models is the confusion matrix. It visualizes the performance of classification models in an $n\times n$ matrix where $n$ refers to the number of classes^30,31^. In addition to confusion matrices, to evaluate the ML and DL models in the classification approach, we used five criteria including accuracy, F1-score, specificity, sensitivity or recall^34,35^, and AUC^28^. These performance metrics are introduced in Eqs. (3–6). The AUC values of the models are expressed according to the study by Mandrekar^29^.

3 $$Accuracy=\frac{TP+TN}{TP+FP+FN+TN}$$

4 $$F1-score=\frac{2\times Precision\times Recall}{Precision+Recall}$$

5 $$Specificity=\frac{TN}{TN+FP}$$

6 $$Sensitivity\vee Recall=\frac{TP}{TP+FN}$$

In Eqs. (3–6), TP, TN, FP, and FN denote True Positive, True Negative, False Positive, and False Negative, respectively.

Three evaluation criteria, including MSE, RMSE, and R^2^, were also used to evaluate the regression models^38–40^. These metrics are explained in Eqs. (7–9).

7 $$MeanSquaredError\left(MSE\right)=\frac{1}{n}\times {\sum }_{i=1}^{n}{\left({Y}_{i}-{\widehat{Y}}_{i}\right)}^{2}$$

8 $$RootMeanSquareError\left(RMSE\right)=\sqrt{\frac{1}{n}\times {\sum }_{i=1}^{n}{\left({Y}_{i}-{\widehat{Y}}_{i}\right)}^{2}}$$

9 $${R}^{2}=\frac{TSS-RSS}{TSS}$$

where $n$ means the number of samples, $i$ is the i-th sample, ${Y}_{i}$ denotes the actual target value for the sample $i$, ${\widehat{Y}}_{i}$ shows the predicted target value for the sample $i$, and $TSS$ denotes the total sum of squares and $RSS$ refers to the residual sum of squares.

### Supplementary Information

Supplementary Information.

## Data Availability

The dataset used in this study can be requested from the SEER source website at https://seerdataaccess.cancer.gov/seer-data-access.
