# Supplementary material for: Survival prediction of glioblastoma patients using modern deep learning and machine learning techniques
Source: Sci Rep. 2024 Jan 29;14:2371. doi: 10.1038/s41598-024-53006-2 (PMC10824760; doi:10.1038/s41598-024-53006-2)
Supplement: Supplementary file 1 — Supplementary Information. [file 41598_2024_53006_MOESM1_ESM.docx]

**Table S1.** Comparison of the significance level of the features.

| *P-*Value < .05 | Feature |
| --- | --- |
| Accepted | Laterality, histologic type ICD-O-3, reason no cancer-directed surgery, age at diagnosis, radiation sequence with surgery, radiation recode, sequence number, year of diagnosis, race recode, chemotherapy recode, primary site, rural-urban continuum code, CS tumor size, insurance recode, summary stage |
| Rejected | Sex, marital status at diagnosis |

**Table S2**. The proposed models’ average performance of each class for the classification approach in five-fold cross-validation strategy.

| Model | Class | Accuracy (%) | F1-score (%) | Specificity (%) | Sensitivity (%) | AUC |
| --- | --- | --- | --- | --- | --- | --- |
| XGBoost | Class 0 | 89.50 | **74.81** | 92.40 | **77.92** | **0.95** |
| AdaBoost | Class 0 | 89.33 | 73.48 | 93.21 | 73.82 | 0.94 |
| DT | Class 0 | 86.57 | 66.86 | 91.28 | 67.75 | 0.80 |
| KNN | Class 0 | 85.96 | 72.40 | 90.15 | 69.21 | 0.81 |
| RF | Class 0 | **89.68** | 74.66 | 93.09 | 76.02 | 0.93 |
| DNN | Class 0 | 88.60 | 69.27 | **95.41** | 62.69 | 0.84 |
| XGBoost | Class 1 | 81.16 | 48.18 | 90.51 | 43.80 | 0.78 |
| AdaBoost | Class 1 | 72.96 | 34.79 | 82.19 | 36.09 | 0.54 |
| DT | Class 1 | 82.55 | 56.19 | 89.22 | 55.93 | 0.74 |
| KNN | Class 1 | 84.62 | 64.25 | 88.51 | 69.10 | 0.85 |
| RF | Class 1 | 87.25 | 66.89 | **92.96** | 64.39 | **0.88** |
| DNN | Class 1 | **88.03** | **70.06** | 91.75 | **73.30** | 0.78 |
| XGBoost | Class 2 | 80.97 | 48.17 | 89.71 | 46.05 | 0.80 |
| AdaBoost | Class 2 | 74.89 | 21.76 | 89.24 | 17.48 | 0.60 |
| DT | Class 2 | 84.80 | 62.26 | 90.33 | 62.67 | 0.78 |
| KNN | Class 2 | 88.25 | 71.02 | 92.31 | 72.01 | 0.89 |
| RF | Class 2 | 89.12 | 72.74 | 93.26 | 72.60 | **0.91** |
| DNN | Class 2 | **90.04** | **75.76** | **93.41** | **77.05** | 0.82 |
| XGBoost | Class 3 | 84.61 | 62.97 | 89.41 | 65.42 | 0.88 |
| AdaBoost | Class 3 | 74.97 | 30.88 | 86.72 | 22.03 | 0.70 |
| DT | Class 3 | 89.02 | 72.51 | 93.17 | 72.38 | 0.85 |
| KNN | Class 3 | 91.96 | 79.43 | **95.53** | 77.66 | 0.93 |
| RF | Class 3 | 92.62 | 81.63 | 95.28 | 81.96 | **0.94** |
| DNN | Class 3 | **92.74** | **82.42** | 94.72 | **84.87** | 0.87 |
| XGBoost | Class 4 | 81.76 | 57.60 | 86.71 | 61.95 | 0.86 |
| AdaBoost | Class 4 | 70.16 | 40.26 | 75.07 | 50.52 | 0.69 |
| DT | Class 4 | 87.65 | 73.63 | 92.60 | 67.80 | 0.82 |
| KNN | Class 4 | 91.12 | 75.08 | **97.19** | 66.84 | 0.92 |
| RF | Class 4 | 91.16 | 78.28 | 94.03 | 79.65 | **0.94** |
| DNN | Class 4 | **91.72** | **79.63** | 94.40 | **80.95** | 0.87 |
| XGBoost | Average | 83.60 | 58.34 | 89.47 | 59.28 | 0.85 |
| AdaBoost | Average | 76.46 | 40.23 | 85.28 | 39.98 | 0.69 |
| DT | Average | 86.11 | 66.29 | 91.32 | 65.29 | 0.79 |
| KNN | Average | 88.38 | 71.43 | 92.81 | 70.97 | 0.88 |
| RF | Average | 89.98 | 74.84 | 93.72 | 74.92 | **0.92** |
| DNN | Average | **90.22** | **75.42** | **93.93** | **75.77** | 0.84 |





**Fig. S1.** AUC diagrams of the DNN model in five-fold cross-validation strategy.





**Fig. S2.** Confusion matrices of the DNN model in five-fold cross-validation strategy.


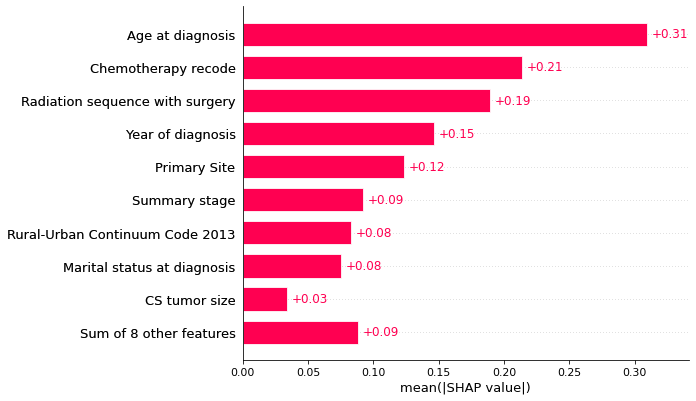


**Fig. S3.** Most important features in DNN obtained using SHAP.


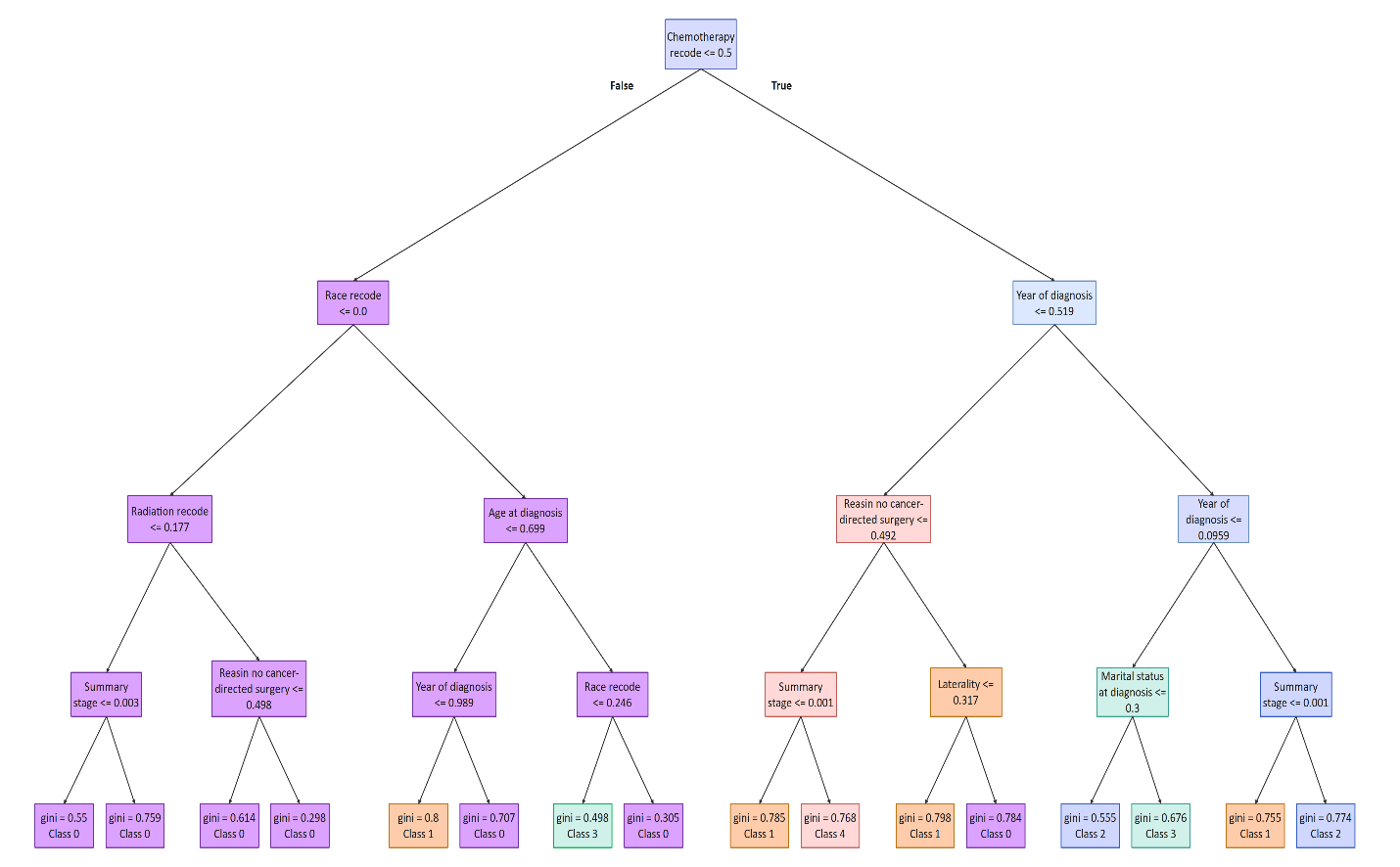


**Fig. S4.** 17th tree of the RF model.


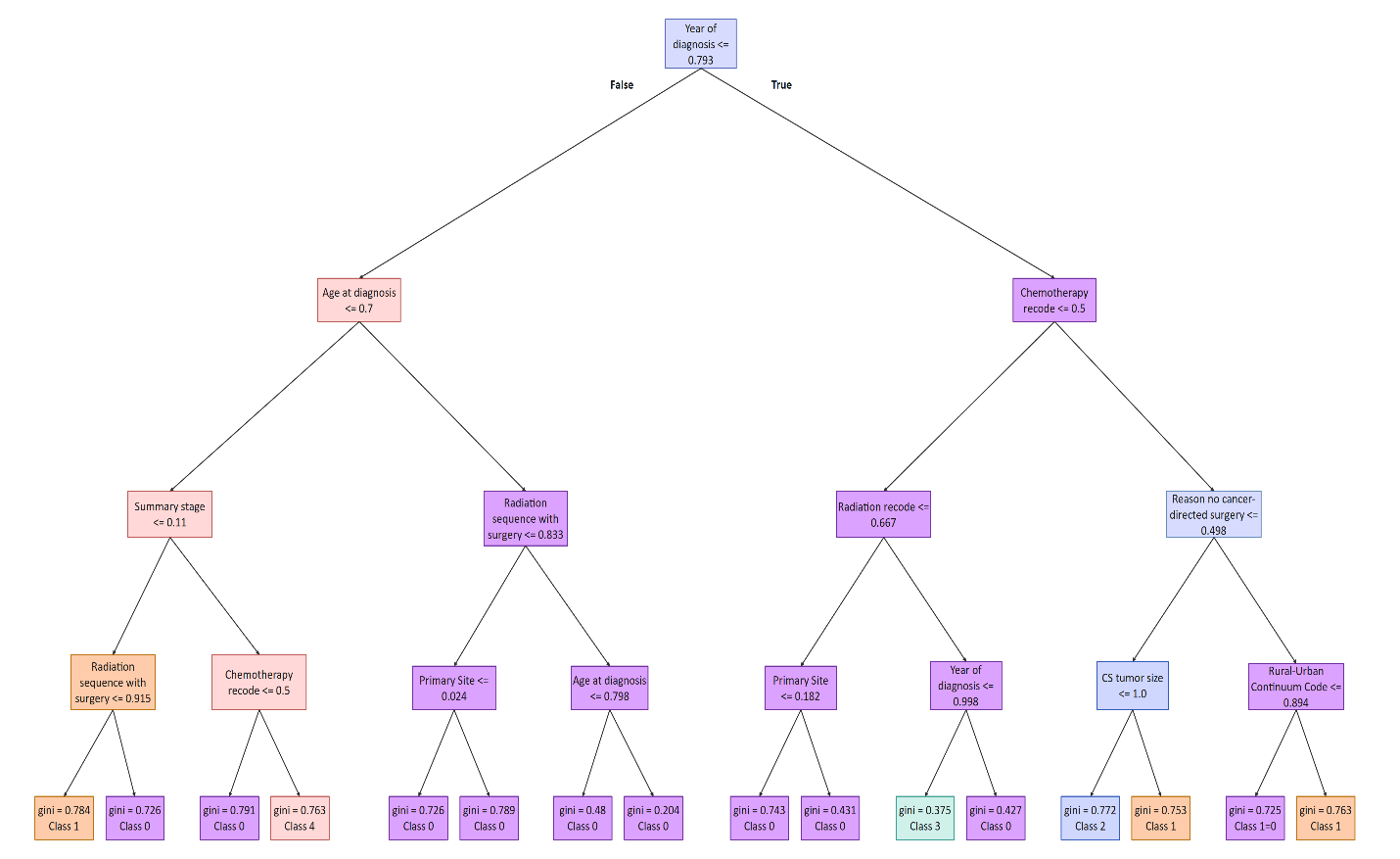


**Fig. S5.** 63rd tree of the RF model.


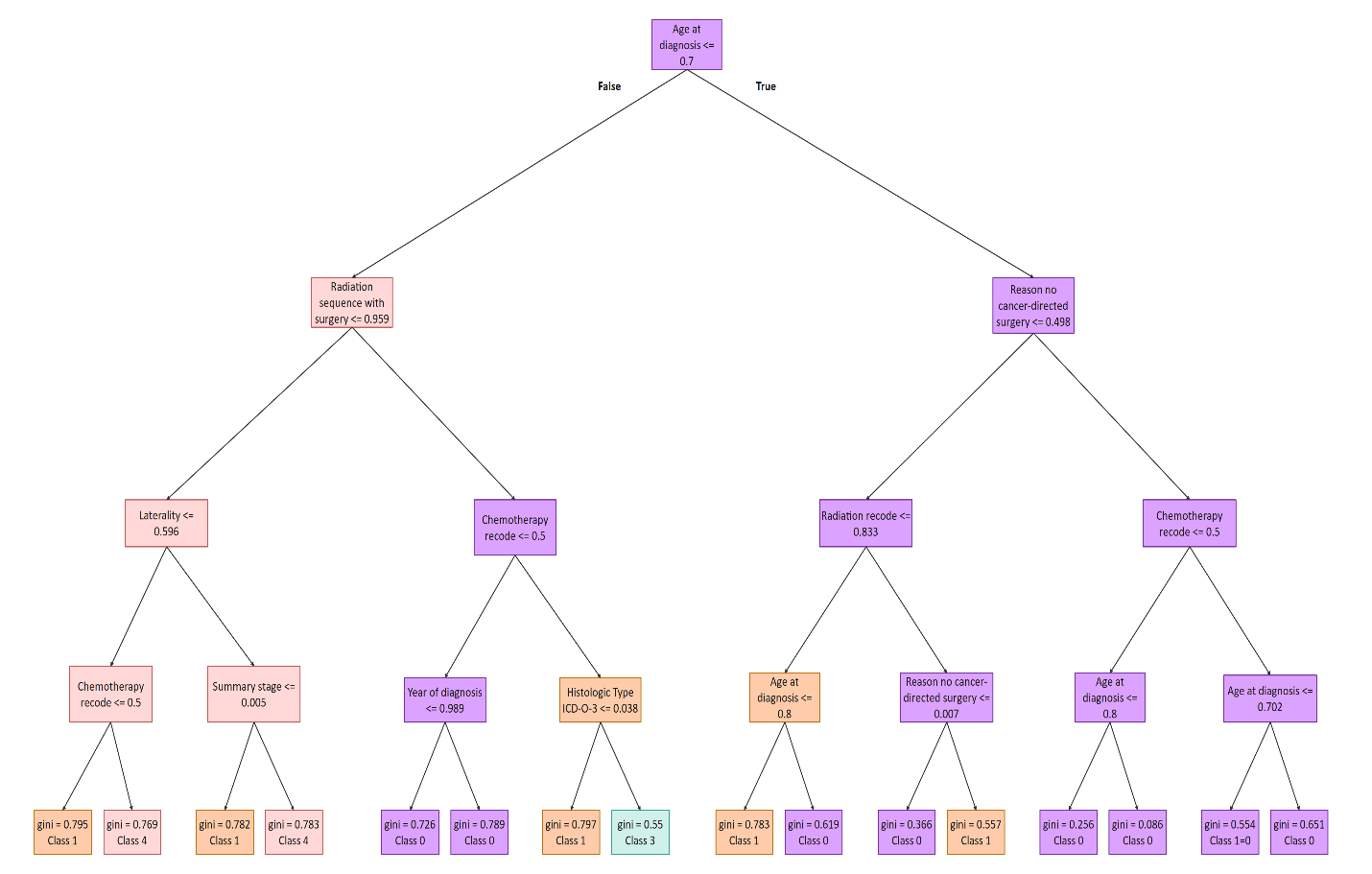


**Fig. S6.** 81st tree of the RF model.

**Table S3.** The proposed models’ average performance for the regression approach in five-fold cross-validation strategy.

| Model | MSE | RMSE (%) | R^2^ |
| --- | --- | --- | --- |
| XGBoost | 0.0170 | 13.06 | 0.6416 |
| AdaBoost | 0.0397 | 20.05 | 0.1773 |
| DT | 0.0216 | 14.73 | 0.5562 |
| KNN | 0.0321 | 17.92 | 0.3434 |
| RF | 0.0172 | 13.16 | 0.6466 |
| DNN | **0.0165** | **12.85** | **0.6622** |

**Table S4.** Dataset description.

| Number | Feature | Type of feature | Domain of values |
| --- | --- | --- | --- |
| 1 | Sex | Categorical | 1. Male 2. Female |
| 2 | Histologic type ICD-O-3 | Categorical | 1. 9440 2. 9441 3. 9442 |
| 3 | Laterality | Categorical | 1. Left - origin of primary 2. Right - origin of primary 3. Bilateral, single primary \| Paired site, but no information concerning laterality \| Paired site: midline tumor 4. Not a paired site, Only one side - side unspecified |
| 4 | Marital status at diagnosis | Categorical | 1. Single (never married) 2. Married (including common law) 3. Divorced \| Widowed \| Separated 4. Unknown |
| 5 | Reason no cancer-directed surgery | Categorical | 1. Surgery performed 2. Not recommended \| Not recommended, contraindicated due to other cond; autopsy only \| Recommended but not performed, unknown reason 3. Unknown; death certificate; or autopsy only \| Recommended, unknown if performed |
| 6 | Radiation sequence with surgery | Categorical | 1. Radiation after surgery 2. Radiation prior to surgery 3. Sequence unknown, but both were given 4. Intraoperative radiation 5. Radiation before and after surgery \| Intraoperative rad with other rad before/after surgery 6. No radiation and/or cancer-directed surgery |
| 7 | Sequence number | Categorical | 1. One primary only 2. 1st of 2 or more primaries |
| 8 | Radiation recode | Categorical | 1. Beam radiation \| Radiation, NOS method or source not specified 2. Radioactive implants \| Radioisotopes 3. Combination of beam with implants or isotopes 4. None/Unknown \| Recommended, unknown if administered \| Refused |
| 9 | Race recode | Categorical | 1. White 2. Black 3. Asian or Pacific Islander 4. American Indian/Alaska Native 5. Unknown |
| 10 | Insurance recode | Categorical | 1. Uninsured 2. Insured \| Any Medicaid \| Insured/No specifics 3. Insurance status unknown \| Blank |
| 11 | CS tumor size | Categorical | 1. 1mm-10mm 2. 11mm-20mm 3. 21mm-30mm 4. 31mm-40mm 5. More than 40mm 6. Unknown \| Blank |
| 12 | Summary stage | Categorical | 1. Regional 2. Localized 3. Distant 4. Unknown/unstaged |
| 13 | Age at diagnosis | Numerical | 11 different age groups (0 to 109) |
| 14 | Year of diagnosis | Numerical | 1. 2007-2009 2. 2010-2012 3. 2013-2016 |
| 15 | Rural-urban continuum code | Categorical | 1. Comp rural lt 2,500 urban pop, adjacent to a metro area 2. Comp rural lt 2,500 urban pop, not adjacent to metro area 3. Urban pop of 2,500 to 19,999, adjacent to a metro area 4. Urban pop of 2,500 to 19,999, not adjacent to a metro area 5. Urban pop of ge 20,000 not adjacent to a metropolitan area 6. Urban pop of ge 20,000 adjacent to a metropolitan area 7. Counties in metropolitan areas of lt 250 thousand pop 8. Counties in metropolitan areas of 250,000 to 1 million pop 9. Counties in metropolitan areas ge 1 million pop |
| 16 | Chemotherapy recode | Categorical | 1. No 2. Yes |
| 17 | Primary site | Categorical | 13 different values |





**Fig. S7.** Distribution of classification dataset after performing SMOTE and regression dataset after performing SMOGN.


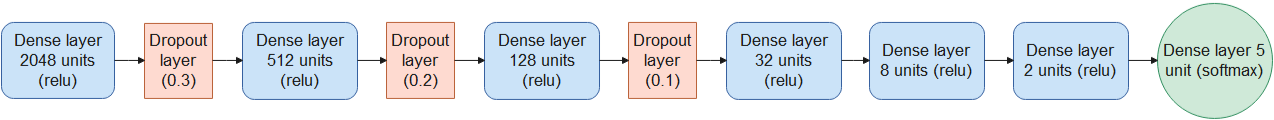


**Fig. S8.** Structure of DNN for the classification approach.


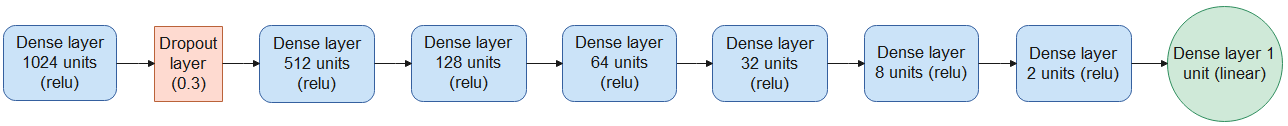


**Fig. S9.** Structure of DNN for regression approach.

**Table S5.** Determined hyperparameters of the models of this study.

| Approach | Model | Hyperparameters |
| --- | --- | --- |
| Classification | RF | max_features: (none, log2, auto, sqrt); criterion: (entropy, gini); n_estimators: (50, 100, 150, 200); max_depth: (none, 5, 15, 25) |
|  | KNN | p: (1, 2); algoritgm: (kd_tree, ball_tree); n_neighbors: (2-20) |
|  | DT | max_depth: (none, 5, 15, 25); criterion: (entropy, gini); max_features: (none, log2, auto, sqrt); spliter: (random, best) |
|  | AdaBoost | learning_rate: (0.5, 0.75, 1, 1.25, 1.5); n_estimators: (50, 100, 150, 200); algorithm: (SAMME.R, SAMME) |
|  | XGBoost | sampling method: (gradiant_based, uniform, subsample); booster: (dart, gblinear, gbtree); max_depth: (3, 6, 9); eta: (0.10-0.95) |
|  | DNN | Number of fully connected layers: 1-7; Number of full connected units: ({2048, 1024, 512, 128, 32, 8, 5}, {2048, 512, 128, 32, 8, 2, 5}, {1024, 512, 256, 128, 64, 32, 5}, {512, 256, 128, 64, 32, 16, 5}, {1024, 256, 64, 16, 8, 2, 5}); Number of epochs: (100, 150, 200, 250, 300); Learning rate: (1e-3, 1e-4, 1e-5); Batch size: (16, 32, 48, 64); Dropout: ({0.3, 0.2, 0.1}, {0.3, 0.15, 0.05}, {0.2, 0.15, 0.1}, {0.2, 0.1, 0.05}, {0.15, 0.1, 0.05}); Loss function: categorical_crossentropy; Last layer’s activation function: softmax; Activation function of layers: (relu, tanh) |
| Regression | RF | max_features: (none, log2, auto, sqrt); criterion: (entropy, gini); n_estimators: (50, 100, 150, 200); max_depth: (none, 5, 15, 25) |
|  | KNN | p: (1, 2); algoritgm: (kd_tree, ball_tree); n_neighbors: (2-20) |
|  | DT | max_depth: (none, 5, 15, 25); criterion: (entropy, gini); max_features: (none, log2, auto, sqrt); spliter: (random, best) |
|  | AdaBoost | learning_rate: (0.5, 0.75, 1, 1.25, 1.5); n_estimators: (50, 100, 150, 200); algorithm: (SAMME.R, SAMME); |
|  | XGBoost | sampling method: (gradiant_based, uniform, subsample); booster: (dart, gblinear, gbtree); max_depth: (3, 6, 9); eta: (0.1-0.9) |
|  | DNN | Number of fully connected layers: 1-8; Number of fully connected layers: 1-7; Number of full connected units: ({2048, 512, 128, 32, 8, 4, 2, 1}, {2048, 1024, 512, 128, 32, 16, 8, 1}, {1024, 512, 128, 64, 32, 8, 2, 1}, {1024, 256, 64, 16, 8, 4, 2, 1}, {512, 256, 128, 64, 32, 16, 8, 1}); Number of epochs: (100, 150, 200, 250, 300); Learning rate: (1e-3, 1e-4, 1e-5); Batch size: (16, 32, 48, 64); Dropout: (0.05, 0.1, 0.15, 0.2, 0.25, 0.3); Loss function: mean_absolute_error; Last layer’s activation function: linear; Activation function of layers: (relu, tanh) |

**Table S6.** Description of the best hyper-parameters of classification and regression models.

| Approach | Model | Hyper-parameters |
| --- | --- | --- |
| Classification | RF | max_features: none, criterion: gini, n_estimators: 100, max_depth: none |
|  | KNN | p: 2, algoritgm: kd_tree, n_neighbors: 2 |
|  | DT | max_depth: none, criterion: gini, max_features: none, splitter: best |
|  | AdaBoost | learning_rate: 1.25, n_estimators: 150, algorithm: SAMME.R |
|  | XGBoost | sampling method: gradient_based, booster: gbtree, max_depth: 6, eta: 0.5 |
|  | DNN | Number of fully connected layers: 1-7, Number of full connected units: 2048, 512, 128, 32, 8, 2, 5, Number of epochs: 250, Learning rate: 1e-4, Batch size: 32, Dropout: 0.3, 0.2, 0.1, Loss function: categorical_crossentropy, Last layer’s activation function: softmax, Activation function of layers: relu |
| Regression | RF | max_depth: none, n_estimators: 150, criterion: gini |
|  | KNN | p: 2, algoritgm: kd_tree, n_neighbors: 4 |
|  | DT | criterion: gini, spliter: best, max_depth: none |
|  | AdaBoost | learning_rate: 0.75, algorithm: SAMME.R, n_estimators: 150 |
|  | XGBoost | booster: gbtree, sampling method: uniform, eta: 0.35 |
|  | DNN | Number of fully connected layers: 1-8, Number of full connected units: 1024, 512, 128, 64, 32, 8, 2, 1, Number of epochs: 250 , Learning rate: 1e-5, Batch size: 32, Dropout: 0.3, Loss function: mean_absolute_error, Last layer’s activation function: linear, Activation function of layers: relu |
